# Translation and validation of the German version of the Systemic Inventory of Change

**DOI:** 10.3389/fpsyt.2025.1686468

**Published:** 2026-01-20

**Authors:** Annina Brendel, Mechthild Hartmann, Markus W. Haun, William M. Pinsof, Beate Wild

**Affiliations:** 1 Department of General Internal Medicine and Psychosomatics, Heidelberg University Hospital , Heidelberg, Germany; 2 Pinsof Family Systems, Chicago, IL, United States

**Keywords:** multisystemic perspective, outcome measure, outpatient therapy, systemic measure, systemic therapy inventory of change (STIC)

## Abstract

**Background and aim:**

The Systemic Therapy Inventory of Change (STIC) is designed to measure changes in family, couple, and individual therapy from a multisystemic and multidimensional perspective. The aim of the present study was to translate the English version of the STIC into German and to evaluate the psychometric properties of the German version in a clinical sample of 309 patients starting outpatient psychotherapy covered by the German Statutory Health Insurance.

**Methods:**

Patients were recruited between July 2023 and November 2024 at Heidelberg Institute for Psychotherapy (HIP) of the University Hospital Heidelberg. In addition to the STIC, several other questionnaires were completed by the participants, including the Patient Health Questionnaire (PHQ-9), Childhood Trauma Questionnaire (CTQ), Experience in Close Relationships (ECR-RD-8), and the Systemic Clinical Outcome and Routine Evaluation (SCORE-15). Pearson correlation coefficients were calculated between the STIC subscales and the corresponding criterion measures.

**Results:**

Significant correlations with various outcome measures between 0.26 and 0.81 demonstrated the construct validity of the German version of the STIC. Multiple linear regression analyses showed that higher scores on the subscale IPS (Individual Problems and Strengths) and RWP (Relationship with Partner) were significantly associated with higher quality of life.

**Conclusion:**

The questionnaire could be used in psychotherapy settings for routine outcome monitoring and psychotherapy research.

## Introduction

In psychotherapy, the focus is primarily on facilitating changes in the client to improve functionality, overall well-being, and to reduce symptoms (1, 2). To quantify improvement or change, many well validated symptom-oriented outcome questionnaires are available (3–5). However, change in systemic therapy may encompass additional aspects. Systemic therapy focuses on individuals and their significant others considering their relationships and broader systems, such as family or social networks. Systemic researchers and clinicians have emphasized the importance of such a multi-systemic perspective in treatment and research (6–8). Questionnaires that focus on individual symptoms will not be sufficient to describe changes in relationships or situations (e.g. 9, 10). Thus, measures that assess change in interpersonal systems are required.

The Systemic Therapy Inventory of Change (STIC; 11) fulfills these conditions and is designed to capture change in family, couple, and individual therapy from a multisystemic and multidimensional perspective. Empirical studies provide support for certain aspects of the STIC Initial, including convergent and discriminant validity of subscale and total scores. Zinbarg et al. (12) reported strong convergent and discriminant validity for most scales, with subscales correlating highly with related measures and weakly with unrelated constructs. Confirmatory factor analyses on aggregated subscale and total scores indicated that the aggregated scale scores reflected broader, higher-order constructs and were largely distinct, supporting the validity in assessing individual, couple, family, and child functioning. Limitations noted by Zinbarg et al. (12) include the lack of suitable validation measures for the Family of Origin (FOO) and Relationship with Child (RWC) scales, as well as untested discriminant validity for some subscales. He et al. (13) found that several STIC Initial scales were sensitive to therapeutic change. The Individual Problems and Strengths (IPS), Relationship with Partner (RWP), and Family/Household (FH) scales were able to detect true clinical change beyond measurement error, whereas the Child Problems and Strengths (CPS) scale did not show evidence of discriminant validity for its change score. Despite these limitations, the study provides preliminary support for the STIC Initial as a measure of multisystemic change from pretreatment to posttreatment and offers additional evidence for its utility in both research and clinical practice.

Beyond measuring change between the beginning and end of therapy, the results from Pinsof et al. (14) suggest that utilizing the STIC as a feedback tool enhances multisystemic outcomes in individual, couple, and family therapy. However, the authors note that a potential limitation could be that they, as the developers of the STIC, conducted the study themselves. In contrast, a study conducted in Norway (15) found no significant differences in therapy outcomes, whether STIC was used as a feedback system or not. This result may be explained by factors related to the design and implementation of the study.

In Germany, systemic therapy gained scientific acceptance in 2008 and formal recognition for reimbursement by health insurance in 2019. Since then, the number of accredited systemic therapists and the caseload of systemic therapies conducted have both been steadily increasing. The STIC is particularly well-suited for evaluating the outcomes of systemic therapy. There are other instruments that focus on systemic aspects, such as the SCORE-15 (16), which evaluates family functioning through three subscales - Strengths and Adaptability, Overwhelmed by Difficulties, and Disrupted Communication - and the ECR-RD8 (17), which assesses partner dynamics. However, these questionnaires target specific domains within an individual’s personal system, whereas the STIC provides a more comprehensive approach, examining multiple levels - including individual, relational, and family functioning - and offering an integrated view of dynamics across these systems. Currently, there is no comparable instrument in Germany that offers such a comprehensive assessment of multiple systems, making the STIC a unique and valuable tool for research and practice in this context. Therefore, the aim of the present study was (1) to translate the English version of the STIC into German and (2) to assess the psychometric properties of the German version. This might enable its future use in Germany for evaluating systemic therapies.

## Methods

### Study design and participants

The study was approved by the ethics committee of the Medical University of Heidelberg (S-014/2023). The STIC was translated from English to German by two systemic therapists from Germany. Subsequently, the German version was back-translated by a bilingual English and German native speaker, blinded with regard to the original version of the STIC, and was then checked for conformity with the original STIC. In the case of inconsistencies between the two versions, the phrasing of the respective item was discussed in a working group of systemic researchers and clinicians. The decision on the most appropriate German translation was achieved by consensus. The final German version of the STIC was applied in the following validation study.

All participants were outpatients recruited at Heidelberg Institute for Psychotherapy (HIP), a large psychotherapy training institution where psychologists receive training in either systemic or psychodynamic psychotherapy. HIP is part of Heidelberg University Hospital and exclusively treats patients with a diagnosed mental disorder, ensuring that all participants were drawn from a clinical population. Treatment at HIP is fully covered by the German health insurance system.

Altogether n=309 patients were included in the study conducted between July 2023 and November 2024. The inclusion criteria of the study were sufficient proficiency in German and written informed consent. Patients completed the STIC along with additional questionnaires at the beginning of the treatment. Participants filled out their demographically matched items, meaning that, for example, a married woman without children filled out the Individual Problems and Strengths (IPS), Family of Origin (FOO), and Relationship with Partner (RWP). Thus, sample size for each scale differed.

### Measures

The Systemic Therapy Inventory of Change (STIC) is assessed from the client’s perspective and consists of the following six scales: Individual Problems and Strengths (IPS), Family of Origin (FOO), Relationship with Partner (RWP), Family/Household (FH), Child’s Problems and Strengths (CPS), and Relationship with Child (RWC). Each scale is subdivided into three to eight empirically developed and validated subscales to assess different subdimensions (e.g., Self-Acceptance and Negative Affect are subscales of IPS; Mutuality of Expectations is a subscale of FOO). The factor structure and psychometric properties of these scales have been supported in clinical samples of therapy clients (11). There are subscales that are positively scored as for example *Life Functioning*, while others are negatively scored, e.g. *Negative Affect.* There are two sets of scales: *the Initial and Intersession.* In this article, we use the term *STIC* to refer specifically to the *STIC Initial*, as only this set was examined in our study. The STIC is completed by the clients before the first therapy session and has a total of 163 questions if the adult has one child; for every additional child, 36 items are added.

For each scale of the STIC, the following questionnaires were used as criterion measures which were completed by the participants alongside the STIC questionnaire:

#### STIC IPS scale

The nine-item depression module of the Patient Health Questionnaire (PHQ-9; 18), a well-validated questionnaire for measuring depression symptoms, was used as a criterion measure for IPS subscales that address symptom severity. Each of the nine items corresponds to one of the DSM-V diagnostic A criteria of a major depressive disorder. Higher PHQ-9 scores indicate greater symptom severity. The Short Form Health Survey (SF-12; 19) was applied to assess health-related quality of life (HRQoL). The SF-12 is a widely used generic questionnaire that does not focus on specific disease groups. Items are weighted and totaled to provide both physical (PCS) and mental component scores (MCS). The instrument includes questions that assess the impact of health on daily activities, physical mobility, vitality, and emotional functioning. Higher scores reflect higher health-related quality of life.

Further, the short version of the OPD-Structure Questionnaire (OPD-SFK; 20) was used to measure personality traits. The OPD-SFK includes 12 items for (self-)assessment of structural personality traits according to the OPD-2, and assesses three structural dimensions: self-perception, contact regulation, and internalized relational models. Higher scores indicate a higher level of structural impairment.

#### STIC FOO scale

The Childhood Trauma Questionnaire (CTQ; 21) was utilized to evaluate adverse childhood experiences. This questionnaire retrospectively measures instances of abuse and neglect occurring before the age of 18. Higher scores on the CTQ suggest greater levels of abuse and neglect.

#### STIC RWP scale

The Experience in Close Relationship (ECR-RD8; 17) questionnaire, which assesses attachment styles (attachment anxiety & attachment avoidance) in adult romantic relationships, was used to validate the RWP scale. The ECR-RD8 provides insight into attachment-related behaviors and feelings of an individual in romantic contexts. Higher scores indicate greater attachment difficulties.

#### STIC FH scale

The Systemic Clinical Outcome and Routine Evaluation (SCORE-15; 16) was included to evaluate family functioning. The SCORE-15 is a proven tool for assessing therapeutic change in family functioning. It evaluates key aspects of family dynamics, emotional distress, and interpersonal relationships, and is divided in three subscales: Strengths and adaptability, overwhelmed by difficulties, and disrupted communication. The higher the score, the more negatively the family was rated.

#### STIC CPS and RWC scales

The Parent version of the Strengths and Difficulties Questionnaire (SDQ-P; 22) was used as a criterion measure for the CPS and RWC scales. It is a brief screening tool designed to assess the mental health and behavioral well-being of children and adolescents from the parent’s perspective. Higher scores on the SDQ Total Scale indicate more behavioral or emotional difficulties, reflecting a lower level of psychosocial functioning.

Sample items for each criterion measure used in this study can be found in Supplement 1.

### Data analysis

Descriptive statistics are provided for the characteristics of the participants and STIC scores. Internal consistency of the STIC was determined by calculating Cronbach’s α.

To assess construct validity, we calculated Pearson’s correlation coefficients between the STIC subscales and the criterion measures described above.

Multiple linear regression analyses were conducted to examine the association between the STIC scales IPS, FOO and RWP and the mental component of HRQoL, measured by the SF-12 subscale MCS. Gender and age were included as control variables.

## Results

### Sample characteristics

The samples for each STIC scale differ slightly, which is why the sample characteristics for each scale are individually reported in **Table 1**.

**Table 1:** Demographic and clinical characteristics of the study sample.

Characteristic	IPS	FOO	RWP	FH	CPS & RWC
*N*	164	309	99	87	45
Gender n (% of N)					
Female	95 (57.9)	196 (63.4)	61 (61.6)	50 (57.5)	26 (57.8)
Male	68 (41.5)	112 (36.2)	38 (38.4)	37 (42.5)	19 (42.2)
Divers	1 (0.7)	1 (0.3)			
Age (years; M ± SD)	40.3 (13.2)	36.9 (12.9)	41.4 (12.1)	40.8 (12.0)	44.8 (7.8)
Education n (% of N)					
College degree	96 (75.0)	189 (67.5)	62 (81.6)	50 (79.4)	27 (60.0)
Advanced degree	18 (14.1)	53 (18.9)	10 (13.2)	7 (11.1)	3 (6.7)
*Missings*	36	29	23	24	12
Married	54 (40.0)	97 (32.6)	49 (61.3)	36 (52.9)	25 (53.3)
*Missings*	29	11	19	19	8
Children					
No children	73 (54.5)	189 (63.9)	39 (48.1)	23 (33.3)	0
≥ 1 child	61 (45.5)	107 (36.1)	42 (51.9)	46 (66.7)	45 (100)
*Missings*	30	13	18	18	
PHQ-9 (M ± SD)	10.7 (5.9)	11.2 (5.7)	11.0 (6.0)	11.0 (5.9)	9.4 (5.9)
GAD-7 (M ± SD)	9.1 (4.9)	9.2 (4.8)	9.7 (5.0)	9.9 (4.7)	8.8 (5.0)
DSM-V Diagnosis n (% of N)					
Affective disorders	121 (73.8)	214 (69.3)	74 (74.7)	60 (69.0)	25 (55.5)
Anxiety disorders	35 (21.3)	70 (22.7)	21 (21.2)	15 (17.2)	4 (8.9)
Somatoform disorders	30 (18.3)	42 (13.6)	22 (22.2)	17 (19.5)	9 (20.0)
Others	79 (48.2)	146 (47.3)	54 (54.5)	42 (48.3)	23 (51.1)
> 1 diagnosis	71 (43.3)	120 (38.8)	49 (49.5)	36 (41.4)	16 (35.6)
Average number of diagnosis (M ± SD)	1.6 (0.9)	1.5 (0.8)	1.7 (0.9)	1.5 (0.8)	1.4 (0.7)

IPS, Individual Problems and Strengths; FOO, Family of Origin; RWP, Relationship with Partner; FH, Family/Household; CPS, Child’s Problems and Strengths; RWC, Relationship with Child; PHQ-9, Patient Health Questionnaire-9; GAD-7, Generalized Anxiety Disorder 7-Questionnaire.

### Internal consistency and mean scores

Cronbach’s Alpha was calculated for all the (sub-)scales to assess internal consistency. The results, along with the sample parameters are provided in **Table 2**; Cronbach’s Alpha for the non-STIC measures is reported in **Supplementary Table S2**. Subscale internal consistencies ranged from 0.03 to 0.95. While most subscales showed good internal consistency, a few subscales had lower alphas. These lower values often corresponded to subscales with few items or items that were rarely endorsed, yet they were retained for their clinical relevance. Further, results show that the mean values of the study fall within the range of the mean values of the clinical sample of Pinsof et al. (23 see **Supplementary Tables S13–S18**).

**Table 2:** Scale mean scores and reliability coefficients.

STIC scale	*k*	*n*	M*	*SD*	*α*
IPS Total	22	164	3.59	0.51	0.88
Flexibility/Resilience	3	164	3.24	0.66	0.64
Life Functioning	2	164	3.08	0.81	0.74
Open Expression	2	164	3.33	0.91	0.57
Self-Acceptance	2	164	3.1	0.99	0.73
Disinhibition*	3	164	4.58*	0.53	0.51
Negative Affect*	6	164	3.23	0.78	0.84
Self-Misunderstanding*	2	164	3.6	1.02	0.7
Substance Abuse*	2	164	4.86	0.33	0.12
FOO Total	22	309	3.83	0.65	0.92
Mutuality of Expectations	2	309	3.68	0.87	0.6
Positivity	6	309	3.53	0.93	0.91
Abuse*	3	309	4.45	0.74	0.64
Intrusiveness*	2	308	3.55	1	0.66
Negativity*	5	309	3.51	0.89	0.84
Substance Abuse*	4	309	4.33	0.66	0.45
RWP Total	24	99	4.17	0.6	0.94
Commitment	2	98	4.18	0.9	0.78
Partner Positivity	9	99	3.85	0.84	0.93
Sexual Satisfaction	2	96	3.66	1.18	0.87
Trust	3	99	4.46	0.78	0.86
Anger/Inequity*	4	99	4.07	0.75	0.74
Physical Abuse*	2	98	4.95	0.17	n/a^†^
Substance Abuse*	2	98	4.75	0.48	0.03
FH Total	28	87	3.93	0.86	0.96
Boundary Clarity	2	86	3.47	0.75	0.04
Decision Making	2	86	3.89	1.02	0.83
Family Pride	2	86	3.83	1.11	0.91
Positivity	9	87	3.84	0.92	0.95
Abuse*	3	86	4.86	0.43	0.74
Feeling Misunderstood*	2	86	3.38	1.21	0.83
Negativity*	8	87	3.98	0.81	0.87
CPS Total	26	45	3.9	0.53	0.9
Parent-Child Alliance	2	50	3.86	0.74	0.78
Prosocial	3	50	3.6	0.64	0.5
Social/Academic	3	51	3.89	0.81	0.62
Antisocial*	6	51	3.98	0.65	0.76
Food/Weight Concerns*	2	50	4.21	1.02	0.84
Impulsivity*	4	49	3.59	0.9	0.79
Negative Affect*	6	51	4.06	0.71	0.85
RWC Total	6	45	3.94	0.66	0.85
Efficacy	2	46	3.72	0.77	0.71
Positivity	2	46	4.16	0.69	0.74
Negativity*	2	46	3.98	0.83	0.69

*k*, number of items; *α*, Cronbach’s alpha; IPS, Individual Problems and Strengths; FOO, Family of Origin; RWP, Relationship with Partner; FH, Family/Household; CPS, Child’s Problems and Strengths; RWC, Relationship with Child.

* Negatively keyed items were reverse-coded so that higher mean scores of all subscales in this table indicate more positive outcomes.

† For this scale, Cronbach’s α could not be computed due to a lack of variance in item responses.

### Construct validity

The IPS subscales showed significant, medium to high correlations (p<0.001) with their respective criterion measures. The highest correlations were between *Negative Affect* and the PHQ-9 (n=149, r=0.76) and between *Self-Acceptance* and OPD-SFK (n=154, r=-0.67). *Substance Abuse* was the only subscale expected to have little to no correlation with our criterion measures, and it was, in fact, the only subscale that showed no significant correlation with the PHQ-9, OPD-SFK or SF-12. This result is consistent with Pinsof et al. (11).

All the FOO subscales showed significant associations (p<0.001) with the CTQ mean scores (n=309), ranging from -0.73 (*Positivity*) to 0.26 (*Substance Use*).

Six of the seven empirically derived RWP subscales correlated significantly (at least p<0.003) with the ECR-RD8 (n=99) mean scores, and varied between 0.31 (*Substance Abuse*) and -0.60 (*Partner Positivity*). *Physical Abuse* was the only subscale that did not show a significant correlation, which was expected, as physical violence is not addressed in the ECR-RD8.

Of the seven empirically based FH subscales, six demonstrated significant correlations with the SCORE-15 (n=87), ranging from 0.45 (Abuse) to -0.81 (Positivity). *Boundary Clarity* did not correlate significantly.

All the CPS subscales showed significant correlations (at least p<0.004) with the SDQ-P mean scores (n=51) ranging from -0.41 (*Parent/Child Alliance*) to 0.78 (*Impulsivity*).

The RWC subscales all correlated significantly (at least p<0.004) in the expected direction with the SDQ-P mean scores (n=46) and ranged from 0.43 (*Negativity*) to -0.54 (*Efficacy*).

All significant correlations were in the expected direction. See all results in **Table 3**. For additional correlations between STIC subscales and non-STIC measure subscales, see **Supplementary Table S3.**

**Table 3:** Correlations between the STIC scales and the criterion measures.

STIC scales	PHQ-9	SF-12 MCS	OPD-SFK	CTQ	ECR-RD8	SCORE-15	SDQ-P
IPS							
Negative Affect	0.76**						
Disinhibition	0.36**						
Life Functioning		0.52**					
Open Expression			- 0.41**				
Flexibility/Resilience			- 0.63**				
Self-Misunderstanding			0.47**				
Self-Acceptance			- 0.67**				
Substance Abuse	0.07	0.06	0.13				
IPS Total	- 0.74**	0.70**	- 0.73**				
FOO							
Mutuality of Expectations				- 0.46**			
Positivity				- 0.73**			
Abuse				- 0.64**			
Intrusiveness				0.48**			
Negativitiy				0.67**			
Substance Abuse				0.26**			
FOO Total				- 0.75**			
RWP							
Commitment					- 0.55**		
Partner Positivity					- 0.60**		
Sexual Satisfaction					- 0.50**		
Trust					- 0.55**		
Anger/Inequity					0.58**		
Physical Abuse					- 0.05		
Substance Abuse					0.31**		
RWP Total					- 0.68**		
FH							
Boundary Clarity						- 0.14	
Decision Making						- 0.62**	
Family Pride						- 0.60**	
Positivity						- 0.81**	
Abuse						0.45**	
Feeling Misunderstood						0.68**	
Negativitiy						0.81**	
FH Total						- 0.86**	
CPS							
Parent/Child Alliance							- 0.41**
Prosocial							- 0.56**
Social/Academic							- 0.62**
Antisocial							0.57**
Food/Weight Concerns							0.45**
Impulsivity							0.78**
Negative Affect							0.73**
CPS Total							- 0.87**
RWC							
Efficacy							- 0.54**
Positivity							- 0.49**
Negativity							0.43**
RWC Total							- 0.58**

PHQ-9, *Patient Health Questionnaire-9*; SF-12 MCS, Short Form Health Survey, Mental Component Score; OPD-SFK, *OPD Structure Questionnaire, short form*; CTQ, *Childhood Trauma Questionnaire*; ECR-RD8, *Experiences in Close Relationships-Revised, short version;* SCORE-15, *Systemic Clinical Outcomes in Routine Evaluation-15*; SDQ-P, *Strengths and Difficulties Questionnaire - Parent version;* IPS, Individual Problems and Strengths; FOO, Family of Origin; RWP, Relationship with Partner; FH, Family/Household; CPS, Child’s Problems and Strengths; RWC, Relationship with Child.

** The correlation is significant at the 0.01 level (two-tailed).

### Regression analyses

Results of the multiple linear regression analyses – controlling for gender and age – indicated that both the IPS (R² = 0.48, p<0.001) and RWP (R² = 0.27, p<0.001) scales were significant predictors of MCS (mental component score of the SF-12), whereas the FOO scale was not (R²=0.10, p=0.822) (see **Table 4** for details). Notably, age was not significantly associated with MCS in the IPS model, when IPS was added as an independent variable to the model. This may be explained by the significant correlation between IPS and age (r=0.25, p=0.001), which may have accounted for the lack of a direct association between age and HRQoL when both variables were included in the model.

**Table 4:** Multiple regression analyses predicting life quality (DV): Model 1 (age + gender + IPS), Model 2 (age + gender + FOO), and Model 3 (age + gender + RWP).

Predictor	*R²*	*B*	*β*	*p*
Model 1	0.48			
Constant		-24.63		<0.001
Age		0.07	0.08	0.275
Gender		-0.53	-0.02	0.745
IPS		15.54	0.66	<0.001
Model 2	0.10			
Constant		21.14		0.012
Age		0.29	0.32	<0.001
Gender		-0.52	-0.02	0.813
FOO		0.39	0.02	0.822
Model 3	0.27			
Constant		-17.60		0.117
Age		0.36	0.35	0.002
Gender		1.82	0.07	0.501
RWP		7.86	0.39	<0.001

DV, dependent variable; *R²*, coefficient of determination; *B*, unstandardized regression coefficient; *β*, standardized regression coefficient; *p*, p-value; IPS, Individual Problems and Strength; FOO, Family of Origin; RWP, Relationship with Partner.

## Discussion

### Key findings

This present study validated the German version of the STIC in a clinical sample of psychotherapy patients treated in a large psychotherapy treatment center in Germany.

The significant correlations between the STIC subscale scores and the PHQ-9, GAD-7, SF-12, OPD-SFK, CTQ, ECR-RD8, SCORE-15, and SDQ-P demonstrate the construct validity of the German version in our study sample. A few subscales (e.g., Substance Abuse, Physical Abuse) were not anticipated to correlate with our criterion measures, and as expected, they did not show a significant correlation with the corresponding measures. Thus, these results support the convergent validity of the STIC scales by showing that subscales expected to correlate with criterion measures do so, while those not expected to correlate show no significant relationships, confirming the scales’ ability to differentiate between related and unrelated constructs.

In the present sample, HRQoL (MCS of the SF-12) was significantly associated with age, but not with gender. Furthermore, the IPS scale showed a strong association with MCS at the beginning of therapy. Individuals reporting less problems and more strengths (IPS) also indicated a significantly higher level of life quality, with a notably high amount of explained variance. Similarly, the RWP scale – assessing the quality of the relationship with one’s partner – was also significantly associated with life quality, although the strength of the association was somewhat lower than for IPS. This finding emphasizes that the quality of the intimate partnership is strongly related to self-perceived quality of life.

In line with earlier findings on the STIC (23), our results show that internal consistency varied substantially across subscales. While most subscales demonstrated acceptable to excellent reliability, a few showed low alpha coefficients (as low as 0.03). This was primarily due to very few items or the assessment of clinically specific behaviors that were rarely present in our sample (e.g., Substance Abuse). Pinsof et al. (23) also reported variability in subscale reliability, although their lowest coefficient was higher (α = 0.39).

### Clinical relevance and applications of the STIC

The large number of subscale scores reflects that the STIC is a complex instrument including a large number of items. However, one of the advantages of the STIC is that it provides a comprehensive assessment of individual functioning, symptoms, well-being, and interpersonal relationships across various systems, such as the couple, the family of origin, and relationships with children. To our knowledge, there is no other questionnaire that combines these aspects in such a comprehensive manner. Additionally, the *STIC Intersession*, a shorter version of the original scale, can be used throughout the course of therapy to monitor progress and changes, which makes it a very practicable tool. While the primary aim of this study was to validate the German translation of the STIC, it is important to emphasize its potential clinical applications. The instrument could be particularly valuable in psychotherapy settings for assessing patients’ individual strengths and challenges, providing insights into areas that may require attention during treatment. Moreover, the STIC could support mental health professionals in tracking patient progress over time and tailoring interventions based on specific domains.

### Limitations

This study has several limitations. Firstly, all data were collected at a psychotherapeutic training institute affiliated with a university hospital, resulting in a clinical sample. Since all participants are patients shortly before psychotherapeutic treatment, this may have led to a certain degree of homogeneity within the sample, particularly in terms of clinical characteristics and psychological distress. This could limit the generalizability of the results to a broader, non-clinical population. Secondly, we did not conduct a factor analysis. The present manuscript already contains extensive information on the psychometric evaluation, and including a detailed factor analytic section would have gone beyond the intended focus of this paper and created an overload of details. Moreover, the STIC has already been validated in its original English version, and our primary goal was to validate the German translation and examine its reliability and construct validity within this new linguistic and cultural context. Given that the factor structure was established in the original version, we considered a confirmatory factor analysis (CFA) not essential at this stage. Instead, we employed correlational analyses and multiple linear regression to explore the relationships between the variables. Nevertheless, we acknowledge that conducting a CFA would be a valuable next step for future research to further confirm the factor structure of the German version.

### Implication

Overall, further research on the STIC in psychotherapy process and outcome studies will be necessary. Having a German version of the STIC now opens up the potential to test this instrument in another country outside of the United States, within the context of systemic psychotherapy.

## Data Availability

The raw data supporting the conclusions of this article will be made available by the authors, without undue reservation.
