# Supplementary material for: Translation and validation of the German version of the Systemic Inventory of Change
Source: Front Psychiatry. 2026 Jan 20;16:1686468. doi: 10.3389/fpsyt.2025.1686468 (PMC12864111; doi:10.3389/fpsyt.2025.1686468)
Supplement: Supplementary file 1 [file Table1.docx]

**Supplement 1**

**Sample Items of the Criterion Measures**

PHQ-9

Over the last 2 weeks, how often have you been bothered by any of the following problems?

- Little interest or pleasure in doing things.
- Feeling down, depressed, or hopeless.
- Thoughts that you would be better off dead or of hurting yourself in some way.

Kroenke, K., Spitzer, R. L., & Williams, J. B. W. (2001). The PHQ‐9: Validity of a brief depression severity measure. Journal of General Internal Medicine, 16(9), 606–613.

SF-12

- How much of the time during the past 4 weeks have you felt calm & peaceful?
- During the past 4 weeks, were you limited in the kind of work you do or other regular activities as a result of any emotional problems (such as feeling depressed or anxious)?
  - Accomplished less than you would like.
  - Did work or activities less carefully than usual

Ware, J. E., Jr., Kosinski, M., & Keller, S. D. (1996). A 12-Item Short-Form Health Survey: Construction of scales and preliminary tests of reliability and validity.

OPD-SQS

- If I think too much about myself, I tend to get confused.
- I don’t have good self-esteem.
- Sometimes my feelings are so intense that I get scared.

Ehrenthal, J. C., Dinger, U., Schauenburg, H., Horsch, L., Dahlbender, R. W., & Gierk, B. (2015). Entwicklung einer Zwölf-Item-Version des OPD-Strukturfragebogens (OPD-SFK)/Development of a 12-item version of the OPD-Structure Questionnaire (OPD-SQS). Zeitschrift für Psychosomatische Medizin und Psychotherapie, 61(3), 262–274.

CTQ

- I felt hated by my family.
- I was physically abused.
- I felt loved.
- I was looked out for.
- My parents were too drunk or high to take care of me.

Bernstein, D. P., Stein, J. A., Newcomb, M. D., Walker, E., Pogge, D., Ahluvalia, T., Stokes, J., Handelsman, L., Medrano, M., Desmond, D., & Zule, W. (2003). Development and validation of a brief screening version of the Childhood Trauma Questionnaire. Child Abuse & Neglect, 27(2), 169–190.

ECR-RD8

- I often worry that my partner will not want to stay with me.
- I find that my partner doesn’t want to get as close as I would like.
- I feel comfortable sharing my private thoughts and feelings with my partner.
- I talk things over with my partner.

Ehrenthal, J. C., Zimmermann, J., Brenk-Franz, K., Dinger, U., Schauenburg, H., Brähler, E., & Strauß, B. (2021). Evaluation of a short version of the Experiences in Close Relationships-Revised questionnaire (ECR-RD8): Results from a representative German sample. BMC Psychology, 9, Article 1.

SCORE-15

- In my family we talk to each other about things which matter to us.
- Each of us gets listened to in our family.
- We trust each other.
- It feels miserable in our family.
- People in the family are nasty to each other.

Stratton, P., Bland, J., Janes, E., & Lask, J. (2010). Developing an indicator of family function and a practicable outcome measure for systemic family and couple therapy: The SCORE. Journal of Family Therapy, 32(3), 232–258.

SDQ-P

Please give your answers on the basis of the child's behaviour over the last six months or this school year.

- Restless, overactive, cannot stay still for long.
- Often has temper tantrums or hot tempers.
- Rather solitary, tends to play alone.
- Generally obedient, usually does what adults request.
- Generally liked by other children.

Goodman, R. (1997). The Strengths and Difficulties Questionnaire: A research note. Journal of Child Psychology and Psychiatry, 38(5), 581–586.

*Note that we used the German version of each instrument for our validation study.*
