# Supplementary material for: Translation and validation of the German version of the Systemic Inventory of Change
Source: Front Psychiatry. 2026 Jan 20;16:1686468. doi: 10.3389/fpsyt.2025.1686468 (PMC12864111; doi:10.3389/fpsyt.2025.1686468)
Supplement: Supplementary file 2 [file Table2.docx]

| **Table S2.**  Reliability Coefficients | | | |
| --- | --- | --- | --- |
| **Criterion measure** | *k* | *n* | *α* |
| PHQ-9 | 9 | 149 | 0.88 |
| OPD-SFK | 12 | 154 | 0.89 |
| CTQ | 25 | 309 | 0.88 |
| ECR-RD8 | 8 | 99 | 0.81 |
| SCORE-15  Strengths and Adaptability  Overwhelmed by Difficulties  Disrupted Communication | 15  5  5  5 | 87  87  86  86 | 0.93  0.89  0.84  0.79 |
| SDQ-P  Emotional Symptoms  Conduct Problems  Hyperactivity  Peer Problems  Prosocial | 25  5  5  5  5  5 | 51  51  51  51  51  51 | 0.78  0.88  0.70  0.86  0.62  0.67 |
| *Note. k* = number of items; *α* = Cronbach’s alpha; PHQ-9 = Patient Health Questionnaire-9; OPD-SFK = OPD Structure Questionnaire, short form; CTQ = Childhood Trauma Questionnaire; ECR-RD8 = Experiences in Close Relationships-Revised, short version; SCORE-15 = Systemic Clinical Outcomes in Routine Evaluation-15; SDQ-P = Strengths and Difficulties Questionnaire - Parent version. | | | |

**Supplement 2**
