# Supplementary material for: Translation and validation of the German version of the Systemic Inventory of Change
Source: Front Psychiatry. 2026 Jan 20;16:1686468. doi: 10.3389/fpsyt.2025.1686468 (PMC12864111; doi:10.3389/fpsyt.2025.1686468)
Supplement: Supplementary file 3 [file Table3.docx]

| **Table S3.**  *Correlations between FH, CPS & RWC and Criterion Measure Subscales* | | | | | | | | |
| --- | --- | --- | --- | --- | --- | --- | --- | --- |
|  | **SCORE-15** | | | **SDQ-P** | | | | |
| **STIC subscales** | Strengths and Adaptability | Overwhelmed by Difficulties | Disrupted Communication | Emotional Symptoms | Conduct Problems | Hyperactivity | Peer Problems | Prosocial |
| **FH** |  |  |  |  |  |  |  |  |
| Boundary Clarity | - 0.19 | - 0.08 | - 0.10 |  |  |  |  |  |
| Decision Making | - 0.66** | - 0.45** | - 0.59** |  |  |  |  |  |
| Family Pride | - 0.64** | - 0.49** | - 0.52** |  |  |  |  |  |
| Positivity | - 0.79** | - 0.69** | - 0.73** |  |  |  |  |  |
| Abuse | 0.42** | 0.39** | 0.41** |  |  |  |  |  |
| Feeling Misunderstood | 0.68** | 0.58** | 0.59** |  |  |  |  |  |
| Negativitiy | 0.77** | 0.70** | 0.73** |  |  |  |  |  |
|  |  |  |  |  |  |  |  |  |
| **CPS** |  |  |  |  |  |  |  |  |
| Parent/Child Alliance |  |  |  | - 0.27 | - 0.38** | - 0.35* | - 0.11 | - 0.37** |
| Prosocial |  |  |  | - 0.54** | - 0.43** | - 0.41** | - 0.13 | - 0.42** |
| Social/Academic |  |  |  | - 0.30* | - 0.47** | - 0.54** | - 0.51** | - 0.45** |
| Antisocial |  |  |  | 0.41** | 0.69** | 0.51** | 0.11 | 0.29* |
| Food/Weight Concerns |  |  |  | 0.47** | 0.27 | 0.28 | 0.25 | 0.30* |
| Impulsivity |  |  |  | 0.55** | 0.62** | 0.85** | 0.27 | 0.41** |
| Negative Affect |  |  |  | 0.85** | 0.44** | 0.45** | 0.35* | 0.36* |
|  |  |  |  |  |  |  |  |  |
| **RWC** |  |  |  |  |  |  |  |  |
| Efficacy |  |  |  | - 0.34* | - 0.60** | - 0.58** | - 0.07 | - 0.32* |
| Positivity |  |  |  | - 0.31* | - 0.46** | - 0.49** | - 0.13 | - 0.36* |
| Negativity |  |  |  | 0.45** | 0.50** | 0.29 | 0.08 | 0.21 |
| *Note.* SCORE-15 = Systemic Clinical Outcomes in Routine Evaluation-15; SDQ-P = Strengths and Difficulties Questionnaire - Parent version; FH = Family/Household; CPS = Child’s Problems and Strengths; RWC = Relationship with Child.  ** The correlation is significant at the 0.01 level (two-tailed).  * The correlation is significant at the 0.05 level (two-tailed). | | | | | | | | |

**Supplement 3**
